# Euglobal-IIIa, a novel acylphloroglucinol-sesquiterpene derivative from *Eucalyptus robusta*: absolute structure and cytotoxicity

**DOI:** 10.1007/s13659-011-0021-9

**Published:** 2011-10-26

**Authors:** Li-Yan Peng, Juan He, Gang Xu, Xing-De Wu, Liao-Bin Dong, Xiu Gao, Xiao Cheng, Jia Su, Yan Li, Qin-Shi Zhao

**Affiliations:** State Key Laboratory of Phytochemistry and Plant Resources in West China, Kunming Institute of Botany, Chinese Academy of Sciences, Kunming, 650201 China

**Keywords:** *Eucalyptus robusta*, acylphloroglucinol-sesquiterpene, absolute structure, cytotoxicity

## Abstract

Euglobal-IIIa (**1**), a novel acylphloroglucinol-sesquiterpene derivative, and a known analogue, have been isolated from leaves of *Eucalyptus robusta*. The structures was elucidated by extensive spectroscopic data and by comparison with data reported in literature, while the absolute configuration of **1** was determined by the X-ray diffraction analysis. Compound **1** exhibited comparable cytotoxicity with that of cisplatin against five human cancer cell lines HL-60, SMMC-7721, A-549, MCF-7, and SW480 with IC_50_ values of 15.7, 15.5, 17.6, 14.3, and 21.8 µM, respectively.

**Electronic Supplementary Material:**

Supplementary material is available for this article at 10.1007/s13659-011-0021-9 and is accessible for authorized users.

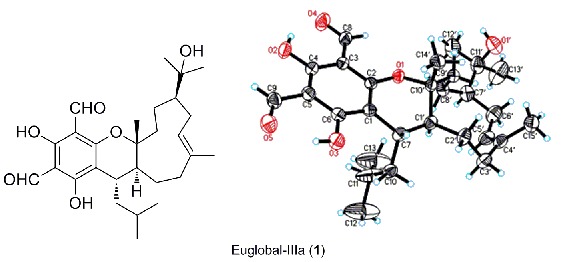

## Introduction

Euglobals are a group of caryophyllene-based monoterpenoid (or sesquiterpenoid) derivatives which have been found to be abundant in the genus *Eucalyptus*.[1] Pharmacological investigations of them showed antitumor, antimicrobial, and granulation inhibiting activities.[2] As part of our efforts to search for significant antitumor agents, a novel acylphloroglucinol-sesquiterpene derivative, named euglobal-IIIa (**1**), and a known analogure, were isolated from leaves of *E. robusta* Smith, a tree up to 20 meters distributed in Yunnan and Sichuan province, China. The structure of **1** was established on the basis of extensive spectroscopic methods and the absolute configuration was determined by the single crystal X-ray diffraction analysis, while the known compound was identified as sideroxylonal B (**2**) by comparison with data reported in literature.[3] The cytotoxicity of two compounds against five human cancer cell lines was evaluated.

## Results and Discussion

An acetone extract of *E. Robusta* was partitioned between $H_2O$ and EtOAc. The isolation of the EtOAc lay afforded a new caryophyllene-based terpenoid, named as euglobal-IIIa (**1**), along with an analogue, sideroxylonal B (**2**).

Euglobal-IIIa (**1**), colorless crystals, was found to posssess a molecular formula of $C_{28}H_{40}O_6$ as assigned by HREIMS at *m/z* 472.2828 $[M]^+$ (calcd. 472.2825 $[M]^+$), implying nine degrees of unsaturation. The UV spectrum showed the existence of a phenyl group based on the maximum absorption bands at 282 and 232 nm, while the FT-IR spectrum exhibited absorption bands for carbonyl groups (1629 $cm^{-1}$) and hydroxy groups (3556 and 3441 $cm^{-1}$).

**1**

**2**

The [1]H NMR spectrum displayed two downfield singlets at $\delta_H$ 10.15 (1H, s) and 9.98 (1H, s) ascribable for two aldehyde groups, an oleanfic signal at $\delta_H$ 5.29 (1H, dd, $J$ = 6.4, 3.2 Hz), six methyl signals (including four singlets at $\delta_H$ 1.07, 1.24, 1.28, and 1.69 and two doublets at $\delta_H$ 0.71 and 0.91) (Table 1). The [13]C NMR spectrum displayed 28 carbon resonances which could be assigned as nine quaternary carbons, seven methines, six methylenes, and six methyls (Table 1). These information suggested that compound **1** possessed three rings. Of the carbon resonances, six quaternary signals at $\delta_C$ 105.7, 169.0, 103.8, 167.8, 104.6, and 163.9, together with two aldehyde carbons at $\delta_C$ 191.8 (d) and 191.9 (d) established an acylphloroglucinol moiety (ring A, Figure 1).[1d,1g,4] Preliminary analysis of [1]H–[1]H COSY spectrum readily established an isopentane group which was connected to C-1 as revealed by the key HMBC correlation of $\delta_H$ 2.68 (1H, m, H-7) with $\delta_C$ 105.7 (C-1), 163.6 (s, C-2), and 169.0 (s, C-6) (Figure 1).

*To whom correspondence should be addressed. E-mail: qinshizhao@mail.kib.ac.cn

Further analyses of $^1$H–$^1$H COSY spectrum established another two partial structures **a** and **b**, which were linked to form the ring C according to HMBC correlations including $\delta_H$ 2.09 (1H, m, H-1') with $\delta_C$ 85.1 (s, C-10') and 36.2 (d, C-7), $\delta_H$ 2.03 (1H, m, H-3′a) and 2.28 (1H, m, H-3′b) with $\delta_C$ 134.4 (s, C-4'), and $\delta_H$ 1.80 (2H, m, H-9′) with $\delta_C$ 85.1 (s, C-10') (Figure 1). In addition, a methyl signal at $\delta_H$ 1.69 (3H, s, H-15') showing HMBC correlations with $\delta_C$ 134.4 (s, C-4') and 41.5 (t, C-3')

**Table 1. $^1$H and $^{13}$C NMR data for 1 ($CDCl_3$, $\delta$ in ppm and $J$ in Hz).[a]**

| No. | $\delta_H$ | $\delta_C$ | No. | $\delta_H$ | $\delta_C$ |
|---|---|---|---|---|---|
| 1 | | 105.7 (s) | 2' | 1.47 m | 32.5 (t) |
| 2 | | 163.9 (s) | 3' | 2.03 m | 41.5 (t) |
| | | | | 2.28 m | |
| 3 | | 104.6 (s) | 4' | | 134.4 (s) |
| 4 | | 167.8 (s) | 5' | 5.29 dd (6.4, 3.2) | 123.9 (d) |
| 5 | | 103.8 (s) | 6' | 2.25 m | 25.1 (t) |
| 6 | | 169.0 (s) | 7' | 1.87 m | 44.7 (d) |
| 7 | 2.68 m | 36.2 (d) | 8' | 1.61 m | 21.2 (t) |
| | | | | 1.81 m | |
| 8 | 10.15 s | 191.9 (d) | 9' | 1.80 m | 33.8 (t) |
| 9 | 9.98 s | 191.8 (d) | 10' | | 85.1 (s) |
| 10 | 1.53 m | 37.9 (t) | 11' | | 73.5 (s) |
| | 2.19 m | | | | |
| 11 | 1.54 m | 25.2 (d) | 12' | 1.24 s | 28.5 (q)[c] |
| 12 | 0.71 d (6.1) | 23.8 (q)[b] | 13' | 1.28 s | 29.0 (q)[c] |
| 13 | 0.91 d (6.1) | 24.3 (q)[b] | 14' | 1.07 s | 21.6 (q) |
| 1' | 2.09 m | 39.8 (d) | 15' | 1.69 s | 16.6 (q) |

[a]Data were assigned based on the HSQC and HMBC spectra.
[b,c]Assignments may be reversed.

suggested the methyl placed at C-4', while the correlation of $\delta_H$ 1.07 (3H, s, H-14') with $\delta_C$ 85.1 (s, C-10') suggested another methyl placed at C-10' (Figure 1). The rest three carbon signals of $\delta_C$ 73.5 (s), 18.5 (q), and 29.0 (q) established a hydroxy substituted isopropyl placed at C-7' as revealed by HMBC correlation of $\delta_H$ 1.87 (1H, m, H-7') with $\delta_C$ 73.5 (s, C-11') (Figure 1). Since compound **1** possessed three ring in the

**Figure 1.** Key 2D NMR correlations of **1** with the X-ray structure showing absolute configuration.

structure as elaborated above and in order to fit the mass unit, there must be an ether bond between C-2 and C-10' to established ring C (Figure 1), which was also supported by the comparison of $^{13}$C NMR data of C-1, C-2, C-7, C-1', C-10' with other analogues.[1a,1d,4] Therefore, the planar structure of **1** was established.

To establish the stereoconfiguration of **1**, an ROESY spectrum was measured. In which, the cross peak between H-7 and H-14' suggested H-7 and Me-14' in the same side, and the cross peak of H-15' with H-6' suggested *E* form of double bond between C-4' and C-5'. However, the stereoconfiguration of C-1' and C-7' could not be determined according to the ROESY spectrum. Finally, an X-ray diffraction not only confirmed the structure of **1** but also established the absolute configuration of the whole molecule (Figure 1).

Both compounds **1** and **2** were evaluated for their cytotoxicity against five human cancer lines using the MTT method as reported previously.[5] Cisplatin was used as the positive control. The results showed that compound **1** displayed comparable cytotoxicity with that of cisplatin against SMMC-7721, A-549, MCF-7, and SW480, while compound **2** was inactive to all the tested strains ($IC_{50}$ > 40 $\mu$M) (Table 2).

**Table 2. Cytotoxicity of 1 and 2 ($IC_{50}$, $\mu$M).**

| | HL-60 | SMMC-7721 | A-549 | MCF-7 | SW480 |
|---|---|---|---|---|---|
| **1** | 15.7 | 15.5 | 17.6 | 14.3 | 21.8 |
| **2** | > 40 | > 40 | > 40 | > 40 | > 40 |
| Cisplatin[a] | 0.9 | 17.1 | 16.0 | 14.9 | 19.1 |

[a]Positive control.

## Experimental Section

**General Experimental Procedures**. Melting points were determined on an X-4 micro melting point apparatus. Optical rotations were measured with a Horiba SEPA-300 polarimeter. UV spectra were obtained using a Shimadzu UV-2401A spectrophotometer. IR spectra were obtained by a Tenor 27 spectrophotometer with KBr pellets. 1D and 2D spectra were run on a Bruker DRX-500 spectrometer with TMS as an internal standard. Chemical shifts ($\delta$) were expressed in ppm with reference to the solvent signals. Mass spectra were recorded on a Waters AutoSpec Primier P776 instrument or an API QSTAR Pulsar i spectrometer. X-ray diffraction was performed on a Bruker SMART APEX-II diffractometer using graphitemonochromated Cu Kα radiation. Column chromatography (CC) was performed using silica gel (200–300 mesh and H, Qingdao Marine Chemical Co. Ltd., Qingdao, People's Republic of China). Fractions were monitored by TLC (GF254, Qingdao Marine Chemical Co. Ltd., Qingdao), and spots were visualized by heating silica gel plates sprayed with 10% $H_2SO_4$ in EtOH. All solvents were distilled prior to use.

**Plant Material.** The leaves of *E. robusta* were obtained from Kunming Botanical Garden, Kunming, China, and identified by Prof. Xiao Chen. A specimen (No. 2009716E) has been deposited at Kunming Institute of Botany, Chinese Academy of Sciences.

**Extraction and Isolation.** An air-dried sample (5 kg) was extracted in acetone at room temperature, and a crude extract was obtained after three times, which was partitioned between $H_2O$ and EtOAc. The EtOAc lay was separated by CC over silica gel (100–200 mesh, Qindao Marine Chemical Ltd., China) eluted with petroleum ether : acetone in a gradient of 0:1

→ 1:1 to afford six fractions (a → e). Fraction b was purified by repeated CC over silica gel (200–300 mesh, Qindao Marine Chemical Ltd., China) eluted with petroleum ether : EtOAc in a gradient of 9:1 → 5:5 to afford five fractions, and euglobal-IIIa (**1**) (20 mg) precipitated from the second one. Compound **2** (50 mg) was obtained from fraction d after CC over silica gel eluted with $CHCl_3$ : MeOH (8:1).

**Euglobal-IIIa (1):** colorless crystals (MeOH); mp 182–184 °C; $[\alpha]_D^{22}$ + 142.9 (*c* 0.05, $CHCl_3$); UV ($CHCl_3$) $\lambda_{max}$ (log $\varepsilon$): 282 (3.93), 232 (3.37), 207 (3.42), 202 (3.42), 192 (3.41); IR (KBr) $\nu_{max}$: 3556, 3441, 2951, 1629, 1442, 1313, 1177, 860 $cm^{-1}$; $^1H$ and $^{13}C$ NMR data, see Table 1; EIMS *m/z* 472 $[M]^+$; HREIMS *m/z* 472.2828 $[M]^+$ (calcd for $C_{28}H_{40}O_6$, 472.2825).

**Crystal data for euglobal-IIIa (1):** $C_{28}H_{40}O_6$, $M$ = 472.60; orthorhomic, space group $P2_12_12_1$; $a$ = 8.11740(10) Å, $b$ = 10.9874 (2) Å, $c$ = 29.3316 (5) Å, $\alpha$ = 90.00, $\beta$ = 90.00, $\gamma$ = 90.00, $V$ = 2616.06 (7) $Å^3$, $Z$ = 4, $d$ = 1.2000 $g/cm^3$, crystal dimensions 0.45 × 0.25 × 0.15 mm was used for measurement on a Bruker SMART APEX-II diffractometer using graphite-monochromated Cu Kα radiation. The total number of reflections measured was 9380, of which 4335, were observed, $I > 2\sigma(I)$. Final indices: $R_1$ = 0.0599, $wR_2$ = 0.1719. Crystallographic data for the structure of **1** have been deposited in the Cambridge Crystallographic Data Centre (deposition number CCDC 809489). Copies of the data can be obtained free of charge from the CCDC via www.ccdc.cam.ac.uk

**Cytotoxicity Assay.** Five human cancer cell lines, breast cancer MCF-7, hepatocellular carcinoma SMMC-7721, human myeloid leukemia HL-60, colon cancer SW480, and lung cancer A-549 cells, were used in the cytotoxic assay. All the cells were cultured in RPMI-1640 or DMEM medium (Hyclone, USA), supplemented with 10% fetal bovine serum (Hyclone, USA) in 5% $CO_2$ at 37 °C. The cytotoxicity assay was performed according to the MTT (3-(4,5-dimethylthiazol-2-yl)-2,5-diphenyl tetrazolium bromide) method in 96-well microplates.[5] Briefly, 100 $\mu$L adherent cells were seeded into each well of 96-well cell culture plates and allowed to adhere for 12 h before drug addition, while suspended cells were seeded just before drug addition with initial density of 1 × $10^5$ cells/mL. Each tumor cell line was exposed to the test compound dissolved in DMSO at concentrations of 0.0625, 0.32, 1.6, 8, and 40 $\mu$M in triplicates for 48 h, with cisplatin (Sigma, USA) as a positive control. After compound treatment, cell viability was detected and a cell growth curve was graphed. $IC_{50}$ values were calculated by Reed and Muench's method.[6]

## Electronic Supplementary Material

Supplementary material is available in the online version of this article at http://dx.doi.org/10.1007/s13659-011-0021-9 and is accessible for authorized users.

## Acknowledgments

This work was financially supported by the National Natural Science Foundation of China (No. 90813004 and No. 2009312311024), the 973 Program (No. 2009CB522300), and the State Key Laboratory of Phytochemistry and Plant Resources in West China (P2010-ZZ05).

**Open Access** This article is distributed under the terms of the Creative Commons Attribution License which permits any use, distribution, and reproduction in any medium, provided the original author(s) and source are credited.

## Electronic supplementary material

Supplementary material, approximately 22.3 KB.
